# Supplementary material for: Evaluation of DNA Extraction Methods Developed for Forensic and Ancient DNA Applications Using Bone Samples of Different Age
Source: Genes (Basel). 2021 Jan 22;12(2):146. doi: 10.3390/genes12020146 (PMC7911526; doi:10.3390/genes12020146)
Supplement: Supplementary file 1 [file genes-12-00146-s001.zip › SupplementaryFigures/SupplementaryFigS1.pdf]

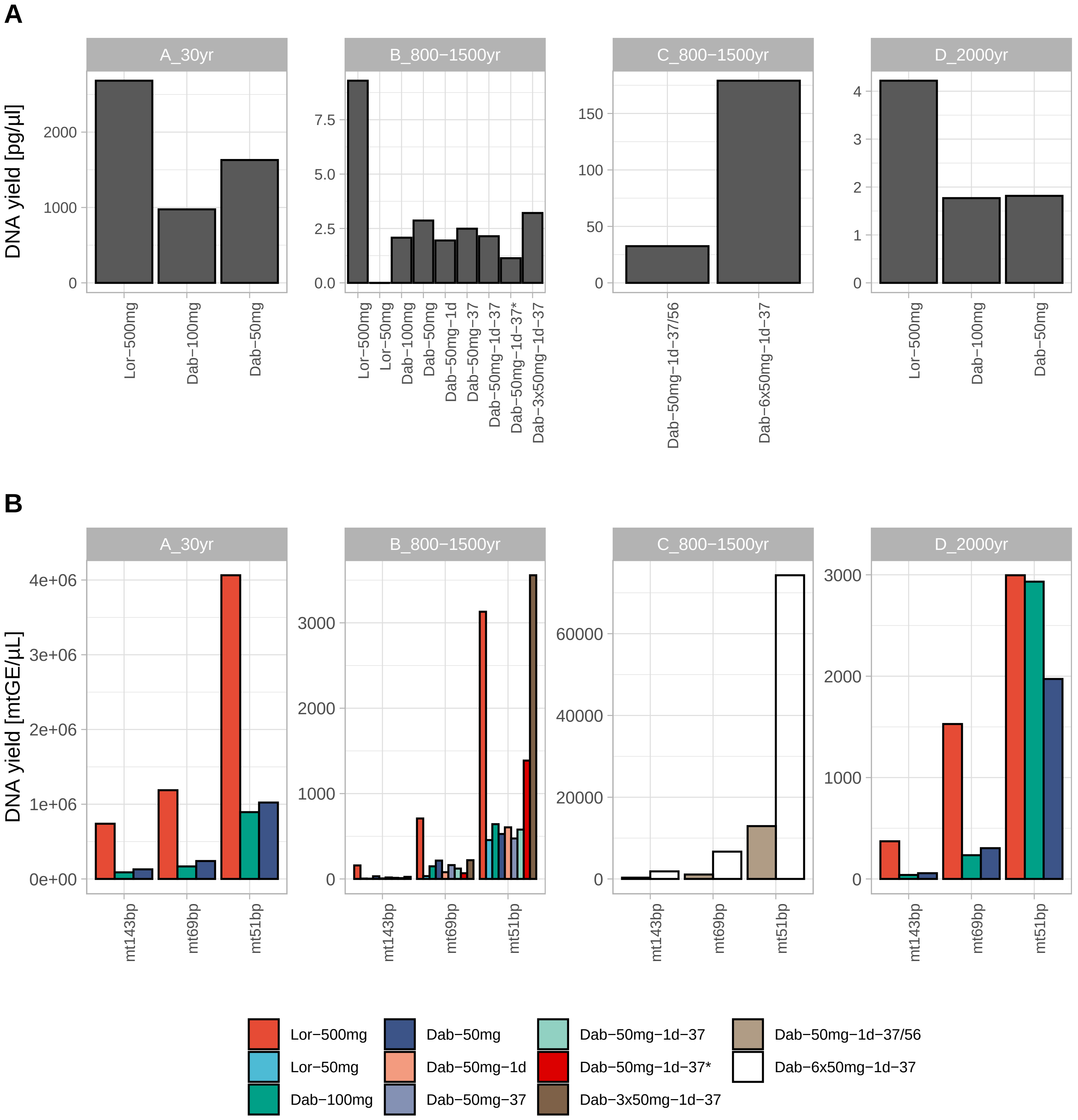

Supplementary figure S1 − Average DNA yield for the nuclear [pg/µl] (A) and mtDNA targets [mtGE/µL] (B) for all extraction trials in all four bone samples tested.
