# Supplementary material for: Evaluation of DNA Extraction Methods Developed for Forensic and Ancient DNA Applications Using Bone Samples of Different Age
Source: Genes (Basel). 2021 Jan 22;12(2):146. doi: 10.3390/genes12020146 (PMC7911526; doi:10.3390/genes12020146)
Supplement: Supplementary file 1 [file genes-12-00146-s001.zip › SupplementaryFigures/SupplementaryFigS10.pdf]

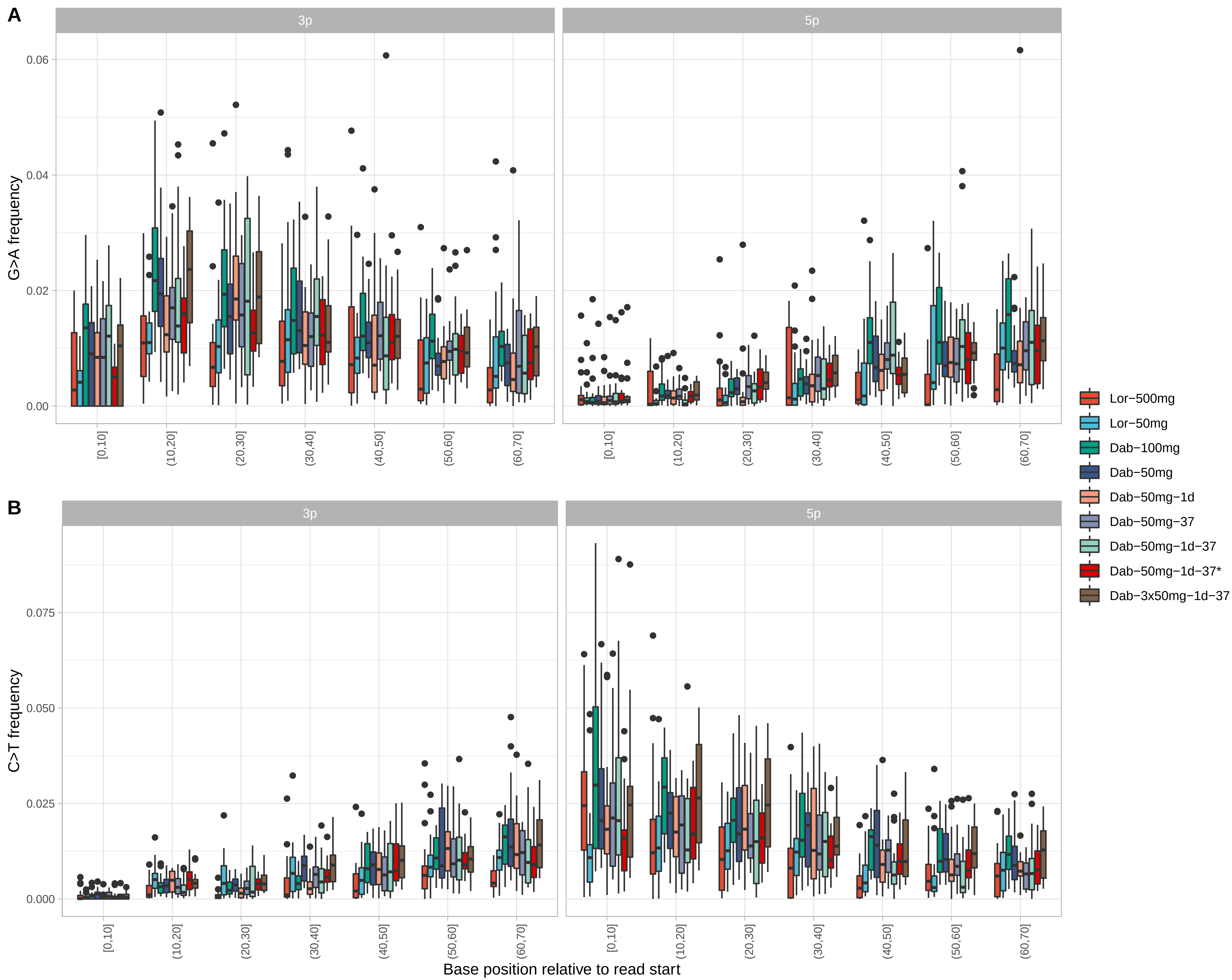

Supplementary figure S10 − Frequency of G>A (A) and C>T (B) damage binned per base position relative to read start in 3p and 5p ends for sample B extraction trials.
