# Supplementary material for: Evaluation of DNA Extraction Methods Developed for Forensic and Ancient DNA Applications Using Bone Samples of Different Age
Source: Genes (Basel). 2021 Jan 22;12(2):146. doi: 10.3390/genes12020146 (PMC7911526; doi:10.3390/genes12020146)
Supplement: Supplementary file 1 [file genes-12-00146-s001.zip › SupplementaryFigures/SupplementaryFigS2.pdf]

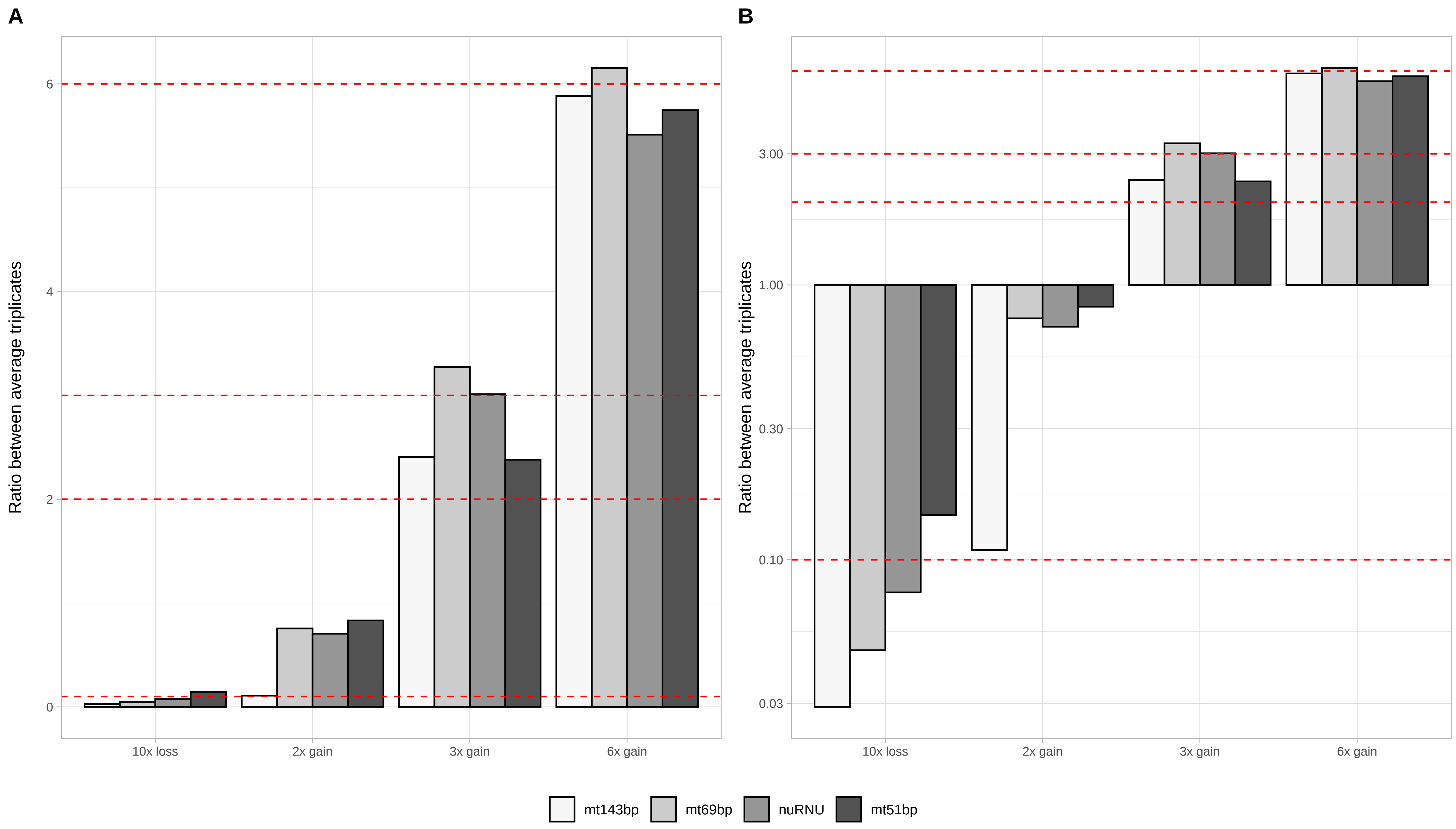

Supplementary figure S2 − Ratio between normal input protocol and alternative protocol (10x loss: Lor−500mg/Lor−50mg, 2x gain: Dab−100mg/Dab−50mg, 3x gain: Dab−3x50mg/Dab−50mg and 6x gain: Dab−6x50mg/Dab−50mg), red lines depict theoretical loss and gain proportions (0.1, 2, 3 and 6). In order to ease visualization both normal coordinates (A) and log 10 scale (B) are presented.
