# Supplementary material for: Evaluation of DNA Extraction Methods Developed for Forensic and Ancient DNA Applications Using Bone Samples of Different Age
Source: Genes (Basel). 2021 Jan 22;12(2):146. doi: 10.3390/genes12020146 (PMC7911526; doi:10.3390/genes12020146)
Supplement: Supplementary file 1 [file genes-12-00146-s001.zip › SupplementaryFigures/SupplementaryFigS3.pdf]

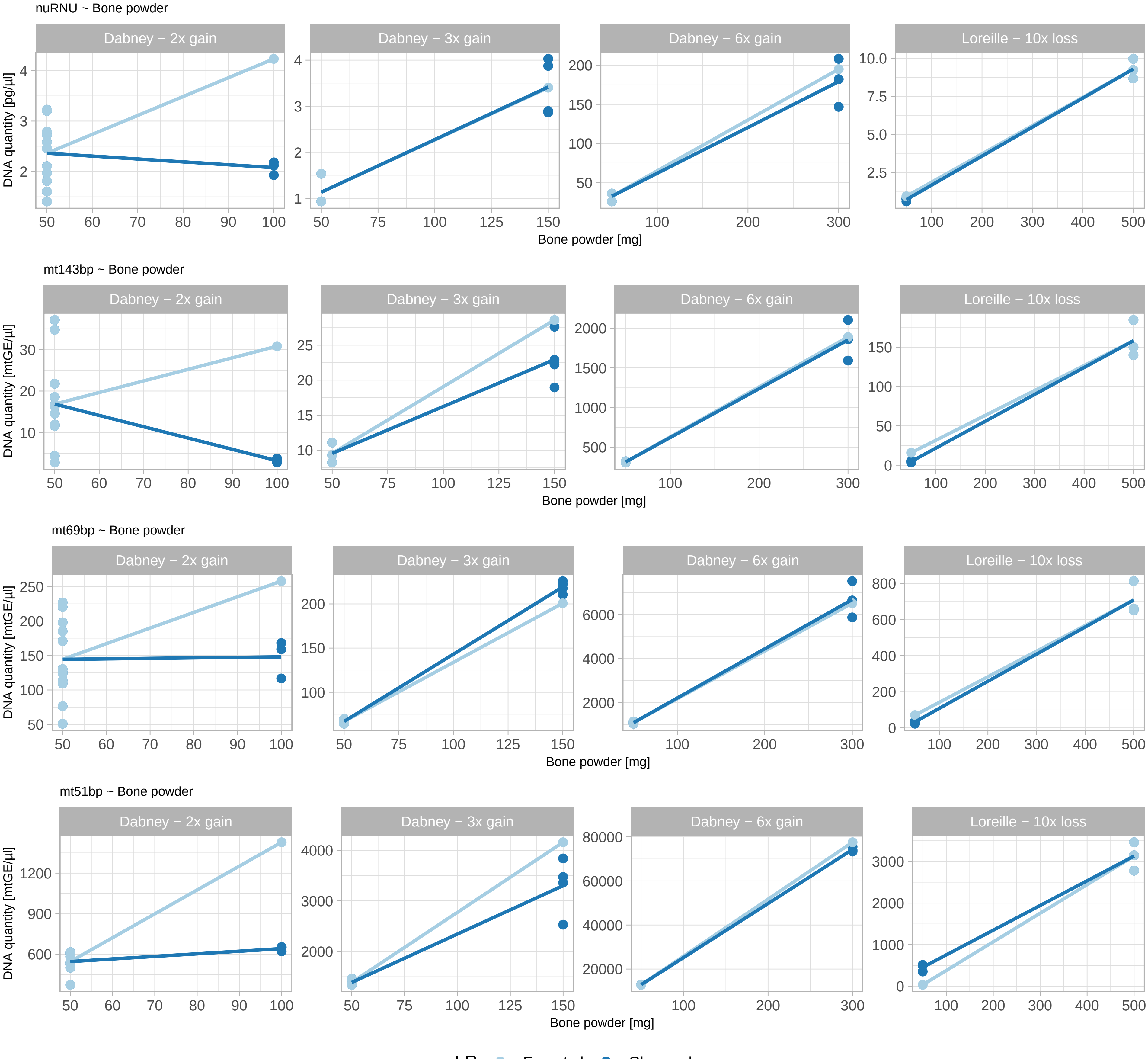

Supplementary figure S3 − Linear regression of DNA yield with weight of bone powder used and expected 10x loss for the Loreille method and 2x, 3x and 6x gain for the Dabney method per quantification target.
