# Supplementary material for: Evaluation of DNA Extraction Methods Developed for Forensic and Ancient DNA Applications Using Bone Samples of Different Age
Source: Genes (Basel). 2021 Jan 22;12(2):146. doi: 10.3390/genes12020146 (PMC7911526; doi:10.3390/genes12020146)
Supplement: Supplementary file 1 [file genes-12-00146-s001.zip › SupplementaryFigures/SupplementaryFigS6.pdf]

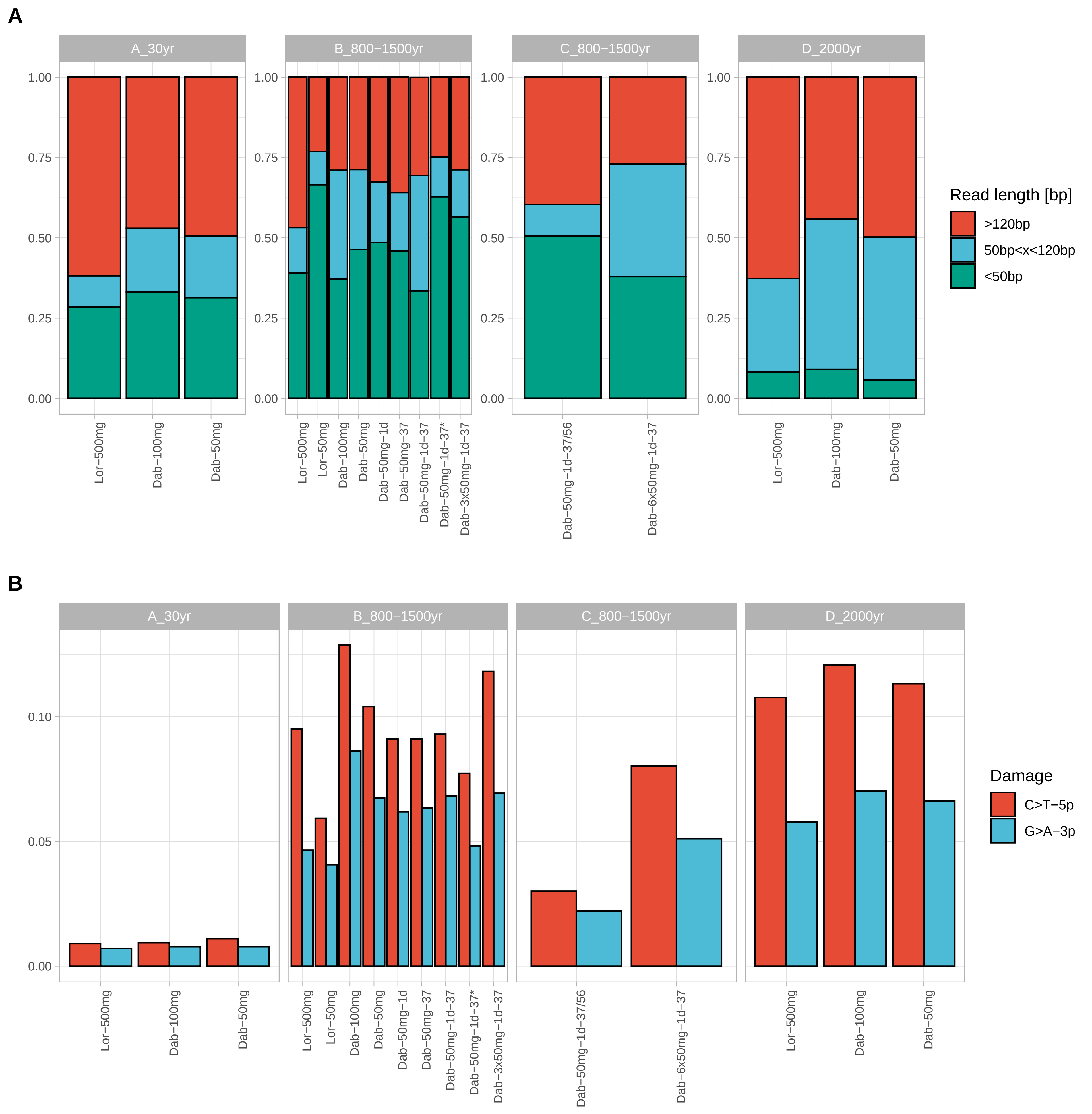

Supplementary figure S6 − (A) Normalized read length distribution per method and sample. (B) Mean frequency of the most expected damage patterns (nucleotide substitutions) in ancient DNA data: G>A at the 3' last 25 positions and C>T at the 5' first 25 positions.
