# Supplementary material for: Evaluation of DNA Extraction Methods Developed for Forensic and Ancient DNA Applications Using Bone Samples of Different Age
Source: Genes (Basel). 2021 Jan 22;12(2):146. doi: 10.3390/genes12020146 (PMC7911526; doi:10.3390/genes12020146)
Supplement: Supplementary file 1 [file genes-12-00146-s001.zip › SupplementaryFigures/SupplementaryFigS7.pdf]

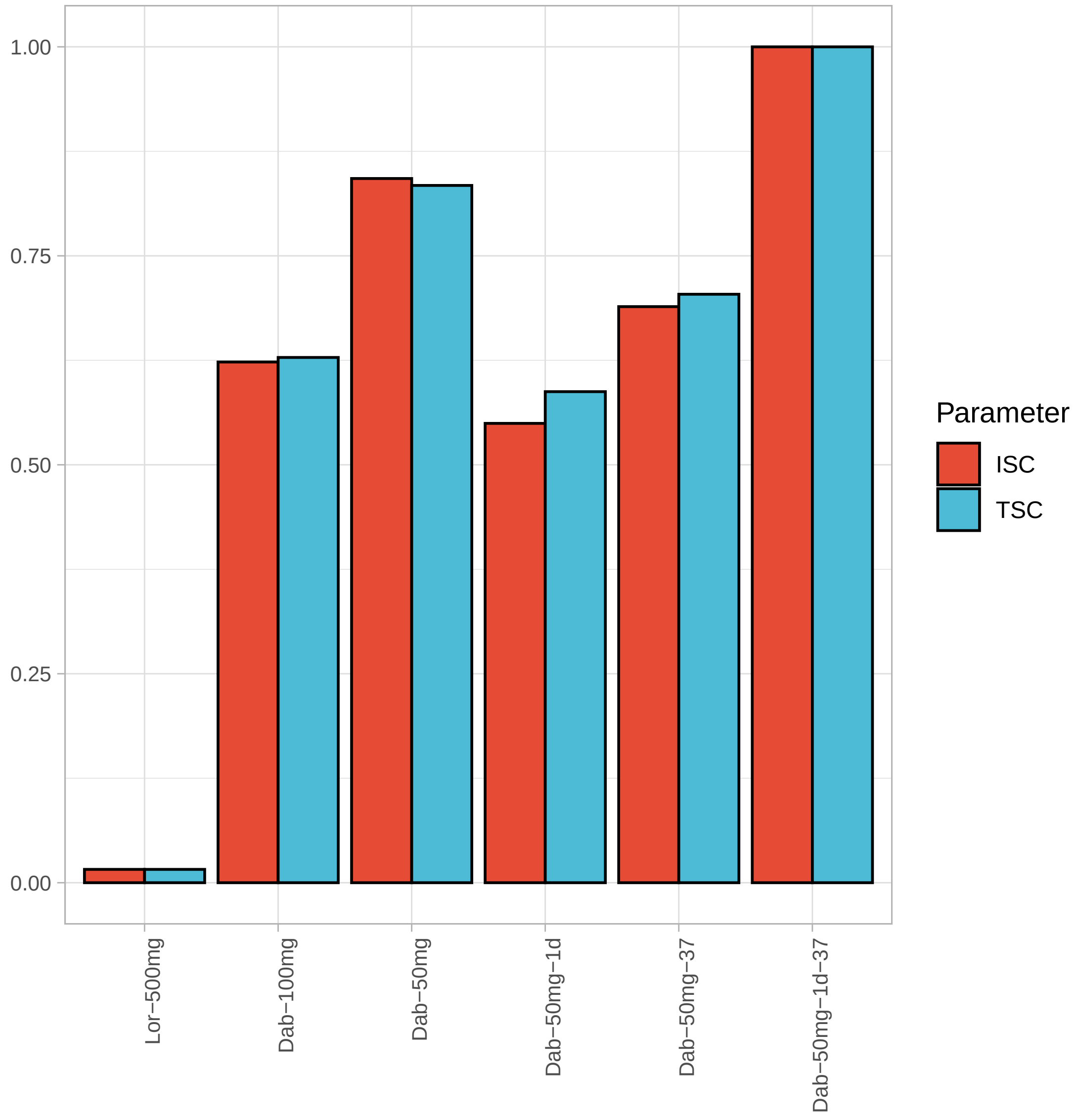

Supplementary figure S7 − Informative and total sequence content (ISC and TSC, respectively) normalized to the best performing method for all earlier trials of Sample B
